# A New Approach To the Diagnosis of Point Mutations in Native DNA Using Graphene Oxide

**Published:** 2016

**Authors:** A.A. Kuznetsov, N.R. Maksimova, V.S. Kaimonov, G.N. Alexandrov, S.A. Smagulova

**Affiliations:** M. K. Ammosov North-Eastern Federal University, Belinskogo str. 58, 677000, Yakutsk, Russia

**Keywords:** point mutations, test system, graphene oxide, diagnosis

## Abstract

Development of new methods for the diagnosis of point mutations is a pressing issue. We have developed a new approach to the design of graphene oxide-based test systems for the diagnosis of point mutations in native DNA. This new approach is based on the use of graphene oxide for the adsorption and quenching of fluorescently labeled primers in a post-amplification PCR mixture followed by detection of fluorescently labeled PCR products. It is possible to detect fluorescently labelled amplicons in the presence of an excess of primers in a PCR product solution due to the different affinities of single-stranded and double-stranded DNA molecules to graphene oxide, as well as the ability of graphene oxide to act as a quencher of the fluorophores adsorbed on its surface. The new approach was tested by designing a graphene oxide-based test system for the DNA diagnosis of the point mutation associated with the development of the 3M syndrome in Yakuts. The developed approach enables one to design graphene oxide-based test systems suitable for the diagnosis of any point mutations in native DNA.

## INTRODUCTION

The diagnosis of point mutations (substitutions, insertions, deletions, hereinafter abbreviated as PMs) is extremely important in modern medicine, since it enables the evaluation of predisposition to various diseases, adequate selection of drugs, and opens the way to the study of genes’ functions. Modern medical genetics uses several basic methods to diagnose PMs in native DNA [1]: PCR-RFLP analysis, fluorescent methods (real-time PCR,end-point PCR), biochip techniques, and sequencing. However, all these methods have certain limitations, and, therefore, discovery of novel approaches to PM diagnosis in native DNA which are faster, more cost-efficient, and effective is a pressing issue [2].

Graphene oxide has two unique properties: quenching of the fluorescence of nearby fluorophores [3] and different affinities to single-stranded and double- stranded DNA molecules [4]. In addition, it is a low-cost and easy-to-synthesize material. For these reasons, it is extensively used in searching for new approaches to PM diagnosis. The use of these properties over the last 5 years has resulted in the development of numerous approaches to PM diagnosis using graphene oxide; for example, [5-9]. However, these approaches are effective in the case of PM diagnosis in short, single- stranded oligonucleotides, and none of them enables PM diagnosis in native DNA [10]. This study was aimed at developing a new approach to PM diagnosis in native DNA using graphene oxide.

## EXPERIMENTAL

**Materials**

Graphene oxide was synthesized from natural graphite powder according to the modified Hummers *et al*. method [11]. We synthesized graphene oxide using reagents supplied by Vostokreaktiv company (Russia), MFPI MF-1230-45 Rusbiolink dialysis bags (Russia), and PCR reagents (PCR buffer, MgCl_2_, dNTP, DNA polymerase) purchased from Evrogen (Russia). We used allele-specific SNPdetect DNA polymerase (Evrogen). The structure of PCR primers is shown in *Table*.

**Table:** Primers for real-time PCR analysis of the material obtained by chromatin immunoprecipitation

Designation	Primer type	Nucleotide sequence, 5’–3’
R	Reverse	GATGAGGCAGTTCAGAAGATTCC
F-FAM	FAM-labeled forward	FAM-CAGGGGTCCTCAAGATTTCG
F-ROX	ROX-labeled forward	ROX-CAGGGGTCCTCAAGATTCG

PCR products were incubated with graphene oxide using sodium phosphate buffer diluted with deionized water (10 ×, Gibco, USA). Deionized water (18.2 MΩ × cm) was obtained using the Advantage A10 Milli-Q purification system (Merck Millipore, Germany).

**Fig. 1:**
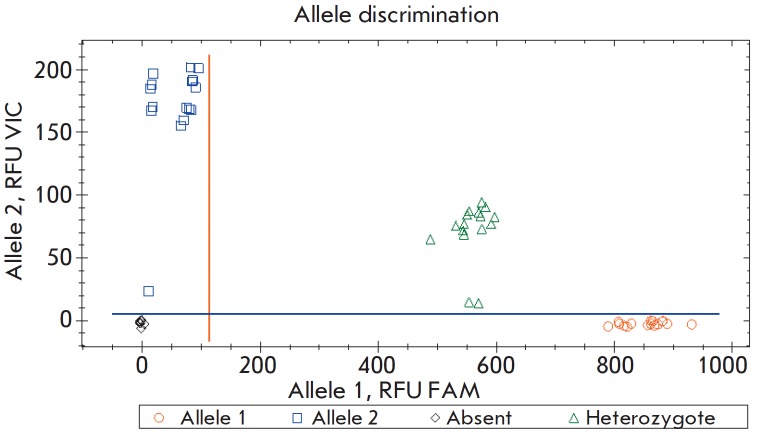
The results of DNA samples genotyping using a real-time PCR test system (FAM channel – 4582InsT mutation, VIC channel – wild type).

The characteristics of the test system were measured in three groups of DNA samples (16 samples in each group, including patients with the 3M syndrome with a confirmed homozygous 4582insT mutation, heterozygous carriers of the 4582insT mutation, and healthy individuals), isolated from the peripheral blood of patients who gave their informed consent. Additionally, 16 negative controls were used. All DNA samples were genotyped using the TestGen test system based on the real-time PCR method (*Fig. 1*). The study was approved by the local ethics committee.

**Equipment**

We used the Intelli-Stirrer MSH-300i magnetic stirrer (Biosan, Latvia), as well as a IL100-6/3 (INLAB, Russia) ultrasonic disperser and MiniSpin Plus centrifuge (Eppendorf, Germany) to synthesize graphene oxide. PCR was performed using a C1000 thermal cycler (Bio- Rad, USA); fluorescence intensity was measured using a Jean-4 fluorometer (DNA-Technology, Russia).

**Graphene oxide synthesis**

Graphite powder (0.1 g, Sigma Aldrich, USA) and sodium nitrate (0.05 g, reagent grade) were added to 14 ml of concentrated sulfuric acid (extra-pure grade). Then, 0.4 g of potassium permanganate (analytical grade) was gradually added in small portions. The resulting reaction mixture was stirred for 3 weeks in a beaker on a magnetic stirrer at 75°C. After stirring, the mixture was diluted to a double volume with deionized water. Further, a 5% hydrogen peroxide solution (7 mL) was added to the mixture until a brilliant-yellow color developed. The brilliant-yellow mixture was filtered using a 70 mm Buchner funnel and ashless filter (yellow band) and washed with 300 ml of deionized water until a neutral pH of the filtrate. This yielded a brown gellike mass, which was transferred from the filter to a beaker and diluted with 50 ml of water, followed by sonication on the IL100-6/3 disperser with a power of 750 W for 5 minutes. After dispersing, the suspension was centrifuged at 14,500 rpm (14.1 g) for 5 min and particles of graphite oxide that were not delaminated by ultrasonic treatment were removed by decantation of the graphene oxide solution above the precipitate. At the last step, the solution was dialyzed in dialysis bags (MWCO: 12000–14000) for 3 days with triple change of deionized water in a 1 liter beaker with the dialysis bag. As a result, 50 mL of a uniform dark brown suspension of graphene oxide was obtained. The atomic proportion of carbon and oxygen was assessed in the dried suspension of graphene oxide by energy-dispersive x-ray spectroscopy and amounted to ~58 and ~42%, respectively. The concentration of graphene oxide in the suspension was determined gravimetrically by weighing a dry residue of 1 ml of the suspension dried at 170°C during 5 min.

**Allele-specific PCR**

For each DNA sample, we prepared 25 μl of the mixture containing 1 × PCR buffer, 3 mM MgCl_2_, 0.28 mM dNTP, 0.2 μM primer R, 0.6 μM primer F-FAM, 66.4 nM primer F-ROX, 2.5 activity units of SNP detect DNA polymerase, and 1.2 ng/μl DNA. The PCR temperature profile consisted of denaturation at 95°C for 3 min, 38 amplification cycles (30 sec denaturation at 95°C, 30 sec annealing at 60°C, 1 min elongation at 72°C), and final elongation at 72°C for 5 min. Amplification was verified by gel electrophoresis of PCR products in 3% agarose gel without ethidium bromide. The length of the amplified product was 149 bps (150 bps in the case of mutant allele amplification); GC-composition was 55.7%.

**Addition of graphene oxide to PCR products and fluorescence measurements.**

We sampled 15 μl of the post-amplification PCR mixture from each tube and placed it into a 0.6 ml transparent microcentrifuge tube. Further, 3.6 μl of 5 × sodium phosphate buffer (Gibco, USA) and a 4 μl of graphene oxide suspension (0.5 mg/ml) in 1 × sodium phosphate buffer (Gibco, USA) were added and incubated at room temperature on an orbital shaker for 20 min (450 rpm). The fluorescence intensity was measured for FAMand the ROX-channels in each tube using a Jean-4 fluorometer (DNA-Technology, Russia).

## RESULTS AND DISCUSSION

**Description of the developed approach**

**Fig. 2:**
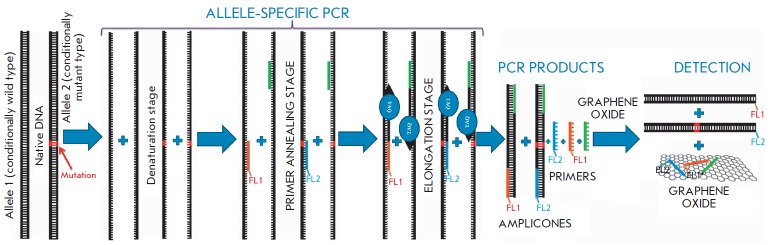
Schematic representation of point mutation diagnosis in native DNA using the developed approach

*Figure 2* shows a schematic diagram of the developed approach.

At the first stage of the diagnosis, we carried out allele- specific PCR, which uses allele-specific DNA polymerase and three primers as opposed to conventional PCR. One of the primers (reverse, highlighted in green in *Fig. 2*) can be complementarily annealed to the DNA of both allele types (wild-type and mutant). Two other primers (forward, highlighted in blue and orange in *Fig. 2*) contain different fluorophores – FL1 and FL2 – with non-overlapping excitation/emission spectra at their 5’-termini. Each of the forward primers can bind to only one type of allele, since they are complementary to the DNA of different alleles at the mutation site.

Depending on the genotype of tDNA donor, three types of the post-amplification mixture can form: with FL1-labeled amplicons (homozygous wild type); FL2-labeled (homozygous mutant type), and with amplicons labeled with both fluorophores (heterozygous type). In either case, the PCR products will contain an excess of fluorescently labeled primers.

When adding the aqueous suspension of graphene oxide to the post-amplification PCR mixture, adsorption of single-stranded DNA molecules, fluorescently labeled primers, will occur on a surface of graphene oxide nanosheets, resulting in quenching of their fluorescence. Double-stranded DNA molecules (amplicons) will remain in solution because of their low affinity to graphene oxide and can generate a fluorescent signal.

The genotype of the DNA donor can be determined by adding an excess of graphene oxide and comparing the fluorescence intensity of each fluorophore in the final solution (for the test DNA sample) and fluorescence intensity in the negative control.

**Testing of the developed approach**

We have developed a test system based on this approach suitable for the DNA diagnosis of the mutation associated with development of the 3M syndrome in Yakuts. The 3M syndrome is a wide-spread autosomal recessive hereditary disease caused by a 4582insT mutation in exon 25 of the *CUL7 *gene (KIAA0076, Cullin- 7) [12]. The 3M syndrome was chosen to develop the graphene oxide-based test system due to the high incidence of heterozygous carriership of the mutation associated with this disease in Yakuts (about 30 individuals per 1,000).

We used the Primer Blast service (http://www.ncbi. nlm.nih.gov) to select ROX- and FAM-labeled primers for different alleles which were complementary to the DNA sequence at the mutation region by 3’-end *(Table)*.

We used the graphene oxide solution (0.5 mg/ml) in 1 × sodium phosphate buffer to add graphene oxide to the PCR products and the buffer alone to neutralize the effect of pH on the fluorescence intensity. The fluorescence intensity of each sample (including negative controls) was then measured for the FAM- and ROX-channels, followed by calculation of the average fluorescence intensity and sample/control intensity ratio in each group of clinical samples for each fluorescence channel individually. Conditions of allele-specific PCR, composition of the PCR mixture, amplified portion length, and amount of graphene oxide were varied to maximize the sample/control intensity ratio for each fluorescence channel.

**Fig. 3:**
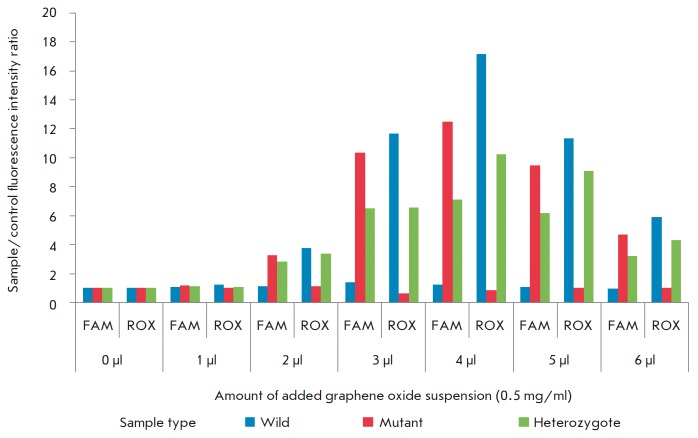
Sample/control fluorescence intensity ratios N for each group of clinical samples for the FAM- and ROX-channels with different amounts of the added graphene oxide suspension.

We optimized the test system using a population consisting of six DNA samples from carriers of the 4582insT mutation and healthy donors (two samples of each type) and two negative controls in seven equivalent experiments with different amounts of the added graphene oxide suspension. In this way, we determined the amount of graphene oxide ensuring the most effective interpretation of the results of the DNA diagnosis, which amounted to 4μl with a concentration of 0.5 mg/ml in 1 × sodium phosphate buffer (*Fig. 3*).

**Fig. 4:**
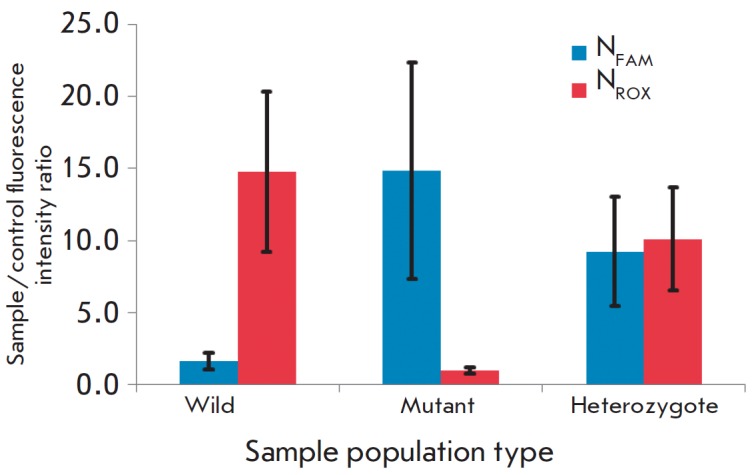
Sample/control fluorescence intensity ratios **N** for each group of clinical samples for the FAM- and ROX-channels.

Testing of the developed test system for genotyping of the control population consisting of 48 DNA samples from carriers of the 4582insT mutation and healthy individuals (16 samples of each type) yielded good results (*Fig. 4*.

The confidence intervals in *Fig. 4* were constructed using standard deviations calculated as the sum of relative standard deviations of the fluorescence intensities of the controls and known samples for each channel.

According to the results shown in *Fig. 4*, the developed test system can reliably diagnose all three combinations of allelic variants in the *CUL7 *gene*.*The use of graphene oxide as a nanostructured fluorescence quencher for fluorescently labeled primers in a post-amplification PCR mixture provided an almost complete fluorescence quenching. At the same time, the fluorescence of the labeled PCR product was largely preserved, which enabled a statistically significant analysis of the post-amplification mixture in terms of its fluorescent properties. The specificity of the test system was 100% in the tested population of clinical samples (since all the samples can be unambiguously attributed to clinical groups), while the sensitivity was no less than 1.2 ng of DNA, which is indicative of the suitability of this approach for the genotyping of point mutations in a conventional genetic laboratory. The apparent advantages of this method are its simplicity (three stages) and rapidity (2 hours). Moreover, in theory, the developed approach is not limited to a specific type of detected point mutations (insertions, deletions, substitutions), since it is based on the use of allele-specific PCR, which enables adaptation of the method to the diagnosis of any point mutation provided that an optimal structure of the primers and optimal diagnostic conditions are selected. Given the simplicity of the method, the low cost of commercial graphene oxide, and availability of the equipment used for DNA diagnostics, the method may be of interest to genetic laboratories involved in pharmacogenetic studies, as well as the diagnosis of genetic diseases caused by DNA point mutations.

## CONCLUSIONS

We have developed an approach that involves the use of graphene oxide as a nanostructured fluorescence quencher for the diagnosis of PMs using allele-specific PCR. The method may be of interest to diagnostic laboratories using inexpensive equipment, such as PCR fluorometers, for the diagnosis of point mutations (substitutions, insertions, deletions) in native DNA. The reliability, specificity, and good sensitivity of this approach were confirmed by the development of a test system for the DNA diagnosis of carriership of the mutation associated with the 3M syndrome in Yakuts. This approach enables one to produce test systems suitable for the diagnosis of any point mutations.

## Abbreviations

PM: point mutation
PCR: polymerase chain reaction
RFLP analysis: analysis of restriction fragment length polymorphisms
FAM: 6-carboxyfluorescein
ROX: carboxy-X-rhodamine
